# Effects of Spermidine on Gut Microbiota Modulation in Experimental Abdominal Aortic Aneurysm Mice

**DOI:** 10.3390/nu14163349

**Published:** 2022-08-16

**Authors:** Shuai Liu, Yu Liu, Jiani Zhao, Pu Yang, Wei Wang, Mingmei Liao

**Affiliations:** 1 Department of General & Vascular Surgery, Xiangya Hospital, Central South University, Changsha 410008, China; 2 Key Laboratory of Nanobiological Technology of Chinese Ministry of Health, Xiangya Hospital, Central South University, Changsha 410008, China; 3 National Clinical Research Center for Geriatric Disorders, Xiangya Hospital, Central South University, Changsha 410008, China

**Keywords:** aortic disease, abdominal aortic aneurysm, spermidine, gut microbiota, polyamines metabolism

## Abstract

Accumulating evidence in recent years has demonstrated the important role of gut microbiota in maintaining cardiovascular function. However, their functions in abdominal aortic aneurysm (AAA) are largely unexplored. In this study, we established a porcine pancreatic elastase-infused experimental AAA mouse model and explored gut microbiota modulation using 16S rDNA sequencing. Here, we found that a significant alteration to gut microbiota composition and function occurred in AAA. The functional change in the gut microbiome revealed dysregulated biosynthesis metabolism and transport of spermidine in AAA. Furthermore, exogenous spermidine was administrated via drinking water and attenuated the progression of experimental AAA disease, which supports our recent study that spermidine alleviates systemic inflammation and AAA. These effects were associated with remitted gut microbiota dysbiosis and metabolism in AAA progression as demonstrated by 16S rDNA gene analysis. In addition, several bacterial florae, such as Bacteroides, Parabacteroides and Prevotella, were identified to be associated with the progression of AAA. Our results uncovered altered gut microbial profiles in AAA and highlighted the potential therapeutic use of spermidine in the treatment of gut microbiota dysbiosis and AAA.

## 1. Introduction

Abdominal aortic aneurysm (AAA) is a potentially fatal vascular disease characterized by the permanent dilation of the aortic wall with a risk of aortic rupture. Recent epidemiologic data indicate that AAA prevalence is around 2.98% among people between 65 and 75 years of age in the United States, posing a high risk to older individuals due to the high morbidity and mortality associated with aortic rupture and dissection. However, no pharmacological approaches have been convincingly proposed to limit AAA growth or to reduce the risk of rupture, highlighting an important need to elucidate the underlying mechanisms of AAA formation and progression [1].

Considerable research has been performed to increase the understanding of AAA pathogenesis, including destruction of the aortic media, progressive and self-reinforcing chronic inflammation and the release of a range of proteolytic enzymes such as matrix metalloproteinases, cysteine proteases, oxidation-derived free radicals, cytokines and related products [2]. Furthermore, bacterial infection was previously proposed as one of the triggers of inflammation in AAA and cardiovascular disease [3]. The first study of microbial detection in AAA was conducted in 1996, indicating a possible role of Chlamydia pneumoniae involvement in the pathogenesis of aortic aneurysms [4]. By employing different techniques, many reports have since shown a potential relationship between the micro-organisms cultured from aortae and the anaerobes in the gastrointestinal tract and oral cavity [5,6,7,8]. Additionally, metabolites of gut microbiota were identified as a contributing factor in maintaining vascular function, including polyamines, trimethylamine-N-oxide, short-chain fatty acids, bile acids and lipopolysaccharide [9,10]. However, although the direct roles of gut microbiota in vascular disorders have been well established as previously reviewed, evidence for the causative role of gut microbiota dysbiosis in AAA disease remains largely unknown [11].

Polyamines are small aliphatic cations with a variety of essential functions in eukaryotes, bacteria and archaea [12]. Spermidine (SPD), a naturally occurring polyamine, has been shown to modulate the gut microbiota and exert a protective effect on cardiovascular diseases [13,14]. Our previous research has demonstrated that oral administration of SPD inhibited the development of experimental AAA by promoting autophagy and inhibiting circulating inflammatory cells [15]. However, the potential mechanism underlying SPD remains unclear in AAA. Given the effect of SPD in modulating gut microbiota, we then aimed to investigate the role and mechanisms of gut microbiota in AAA development. In this study, we will discuss the alteration to gut microbiota in AAA, and whether the modulation of gut microbiota by exogenous SPD can be used for potential therapeutic purposes.

## 2. Materials and Methods

### 2.1. Porcine Pancreatic Elastase (PPE)-Induced AAA Model in Mice

Ten-week-old male C57BL/6 mice were purchased from the Xiangya School of Medicine, Central South University. AAAs were created via transient intra-infrarenal aortic infusion of PPE, as previously detailed [15]. Briefly, mice were anesthetized with inhaled isoflurane and surgical procedures were performed under sterile conditions. After exposing the infrarenal aorta, the aorta was cannulated with a PE-10 polyethylene microcatheter and subjected to transient infusion of type I PPE (4.0 U/mL; E1250, Sigma-Aldrich, St. Louis, MO, USA) dissolved in saline at a pressure of 150 mm Hg. Nylon sutures (10-0) were used to close the aortotomy after infusion. The aortic diameters of pre-infusion and post-infusion were measured to ensure consistency and reproducibility for each group [16]. Following the closure of the laparotomy, mice were housed under specific pathogen-free conditions in a 12-h light/dark cycle with ad libitum access to food and water.

SPD (3 mM, S0266, Sigma Aldrich) was administrated via drinking water, as previously reported [15]. Spermidine, at this concentration, promotes longevity [17] and cardioprotection [18], as well as reverses atherosclerosis [19] and arterial aging [20]. The mice were randomly divided into 4 groups: sham group (n = 4) and SPD group (n = 4) were both infused with saline as control; PPE group (n = 13) and PPE-SPD group (n = 10) were both infused with PPE to establish AAA model. 

### 2.2. Imaging AAA Formation and Progression

Aortic aneurysmal enlargement was monitored by measuring the aortic intro-diameter using serial transabdominal high-frequency ultrasound (Vevo^®^ 2100 Imaging System, VisualSonics, Toronto, ON, Canada) and extra-diameter using a digital camera under laparotomy. Measurements of aortic extra-diameter were performed before and after perfusion on day 0, and before sacrifice on day 14. Measurements of aortic intro-diameter were performed on day 0 (hours before infusion, pre-infusion), day 1 (the day after infusion, post-infusion), day 3, day 7 and day 14 by investigators blinded to group assignment. 

### 2.3. Histological Analyses

Mice were sacrificed 14 days after PPE infusion. Aortae were harvested, fixed in 4% paraformaldehyde, embedded in paraffin, and horizontally cut into sections. For histological analyses, hematoxyline-eosin (H&E) staining, elastic Van Gieson (EVG) staining and a 2-step standard immunoperoxidase procedure for immunohistochemistry were conducted to identify elastic fiber fragmentation and medial smooth muscle cell destruction. 

### 2.4. Fecal Sample Collection and DNA Extraction

Cecal contents (300 mg) of mice were collected 14 days after PPE infusion for 16S rDNA sequencing. Cecal contents were kept in 5 mL tubes with 2 mL preservative buffer (0.5 mol/L Tris, 0.15 mol/L EDTA, 10 mmol/L NaCl, pH 9.0). DNA was extracted using the QiaAMp DNA Stool Mini Kit (QIAGEN, Hilden, Germany) according to the manufacturer’s instructions. DNA concentrations were determined using the Qubit quantification system (Thermo Scientific, Waltham, MA, USA). Extracted DNA was stored at −20 °C.

### 2.5. 16S rDNA Gene Analysis

Bacterial DNA extraction, PCR amplification and sequencing of 16S rDNA gene were carried out by GeneTalks Biotech Co., Ltd. (Changsha, China). For each sample, the V3-V4 region of the 16S rDNA gene was sequenced with the Illumina MiSeq/HiSeq2500 high-throughput sequencing platform. Bioinformatic analysis was performed with QIIME2 after sequence quality control and denoising using fastp software. The Kyoto Encyclopedia of Genes and Genomes (KEGG) pathway analysis was performed https://www.genome.jp/kegg/pathway.html (accessed on 30 April 2022). Sequencing data are available at NCBI under the BioProject ID PRJNA823834.

### 2.6. Statistical Analysis

All data are expressed as means ± SEM. Statistical analyses were determined using R v3.5.0 and Graphpad Prism. Student’s *t*-test, Kruskal–Wallis test, Wilcoxon rank-sum test and one-way ANOVA were used to determine the difference between different groups. Correlations between AAA diameters and the gut microbiome were decided based on Pearson’s correlation coefficients. A *p*-value ≤ 0.05 was considered statistically significant.

## 3. Results

### 3.1. The Gut Microbiota Diversity Changed in PPE-Induced Experimental AAA

**Figure 1:**
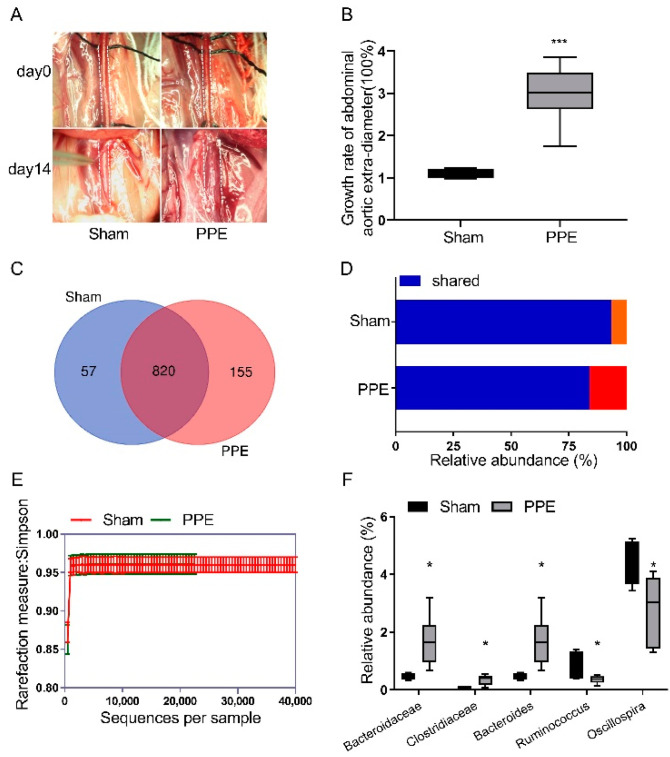
The gut microbiota diversity changed in PPE-induced AAA disease. (**A**) Representative images of experimental AAA at day 0 and day 14. (**B**) The growth rate of abdominal aortic external diameter in sham (n = 4) and PPE (n = 6) groups. Data were presented as mean ± SEM. Unpaired *t* -test, ***, *p* < 0.001 vs. sham group. (**C**) The numbers of OTUs in sham group, PPE group and shared OTUs in both groups in a scaled Venn diagram. (**D**) Total abundance of OTUs in sham and PPE groups. (**E**) Alpha diversity measured by Simpson rarefaction curve. (**F**) Relative abundance statistical analysis between sham and PPE groups. Data are presented as mean ± SEM. Unpaired *t*-test, *, *p* < 0.05 vs. sham group. PPE, porcine pancreatic elastase. AAA, abdominal aortic aneurysm. OTUs, operational taxonomic units.

PPE or saline was intraluminally infused to establish experimental AAA, and a sham group acted as a control (Figure 1A). The maximal external abdominal aortic diameter increased threefold on average after PPE infusion, with increased aortic destruction compared to the sham group (Figure 1B and Supplemental Figure S1A). To investigate the alteration in the gut microbiome in AAA, DNA samples from cecal contents were collected to conduct 16S rDNA analysis. A total of 877 operational taxonomic units (OTUs) were obtained in the sham group, while 975 OTUs constituted the gut microbiota in the PPE group (Figure 1C). Out of these, 820 OTUs constituted the common microbiota which was at lower relative abundance in the PPE group (Figure 1D). Moreover, a lower alpha diversity (measured by Simpson rarefaction curve) was observed in the PPE group as compared to the sham group (Figure 1E). These results suggest the dysbiosis of the gut microbiota and decreased gut microbiome diversity in AAA. 

Based on the OTU abundance and taxonomic annotation in the KEGG database, we investigated the differences in gut microbiota at the phylum/order/class/family/genus taxonomic level (Supplemental Figure S1B–F). According to these results, relative abundance statistical analysis suggested Bacteroidaceae, Bacteroides and Clostridiaceae increased, while Ruminococcus and Oscillospira decreased in the PPE group at the family/genus level (Figure 1F).

### 3.2. Functional Alterations in Gut Microbiota in Experimental AAA

**Figure 2:**
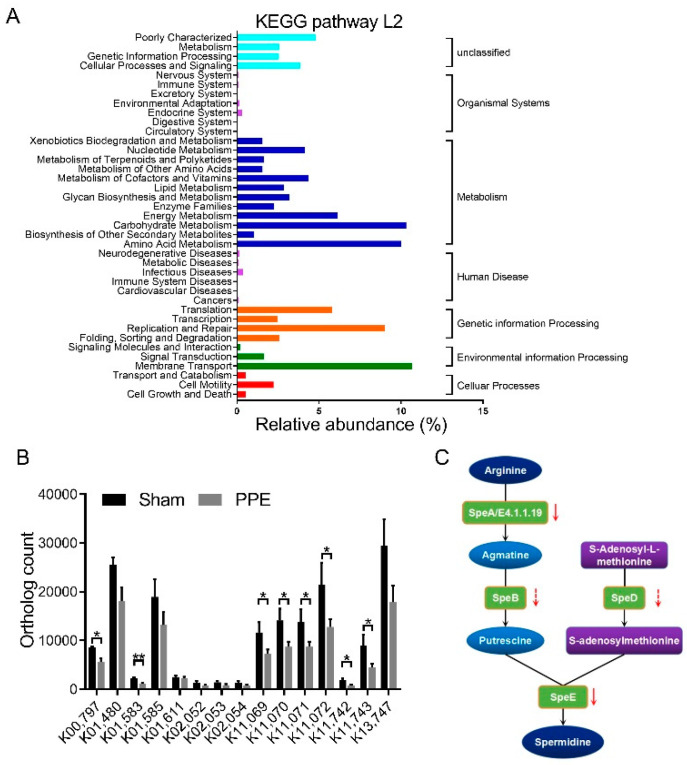
Functional alterations in gut microbiota in experimental AAA. (**A**) KEGG pathway L2 and gene relative abundance detected in the PICRUSt inferences. The related pathway annotations of each column are listed on the left, and related names of KEGG pathway L1 are listed on the right. (**B**) Ortholog count of indicated KOs in sham and PPE groups. Data are presented as mean ± SEM. Unpaired *t*-test, * *p* < 0.05, ** *p* < 0.01, vs. sham group. The name of indicated KOs and involved enzymic reactions are shown in Supplemental Table S1. (**C**) Schematic diagram of the biosynthesis of SPD and polyamine metabolism. AAA, abdominal aortic aneurysm. KEGG, Kyoto Encyclopedia of Genes and Genomes. KOs, KEGG Orthologs.

Next, we investigated the functional change in the gut microbiome in the progression of AAA. KEGG pathways and gene relative abundance were detected in the PICRUSt inferences (Figure 2A). At KEGG level 2, carbohydrate and amino acid metabolism, as well as membrane transport increased in experimental AAA. LEfSe analysis suggested the biosynthesis metabolism and transport system of polyamine, especially SPD, were remarkably reduced in the PPE group (Figure 2B and Supplemental Table S1). Since commensal gut bacteria was an important source of systemic SPD, dysregulation of SPD metabolism in gut microbiota may play an important role in the progression of AAA, as described in the KEGG pathway annotation in Figure 2C.

### 3.3. Effect of SPD on Experimental AAA and Gut Microbiota Diversity

**Figure 3:**
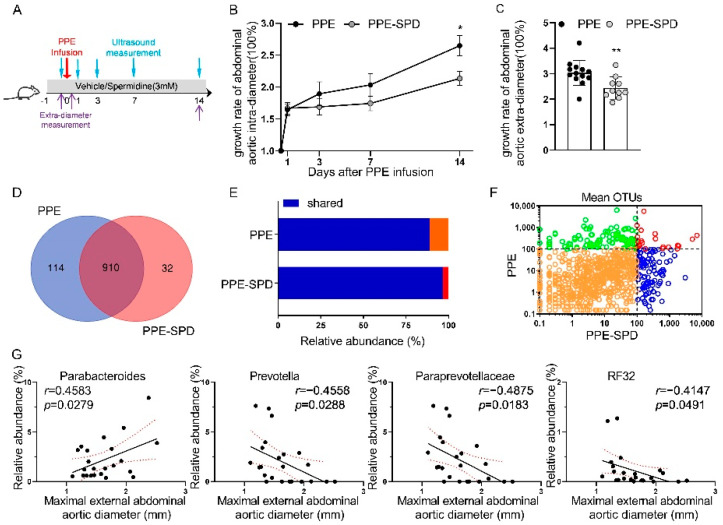
Effect of SPD on AAA progression and gut microbiota modulation. (**A**) Detailed schematics for the experimental AAA models used in this study. (**B**,**C**) The growth rate of abdominal aortic intra-diameter and extra-diameter, respectively, for the experimental AAA models of PPE group (n = 13) and PPE-SPD group (n = 10). Data are presented as mean ± SEM. Significance was analyzed using two-way ANOVA with the Bonferroni correction and unpaired *t*-test, respectively. * *p* < 0.05; ** *p* < 0.01 vs. PPE group. (**D**) The number of OTUs in PPE group only, PPE-SPD group or shared between two groups in a scaled Venn diagram. (**E**) Total abundance of OTUs in two groups. (**F**) Distribution of mean OTUs in two groups. (**G**) Correlations between the gut microbiome and the maximal external abdominal aortic diameter based on Pearson’s correlation coefficients. AAA, abdominal aortic aneurysm. PPE, porcine pancreatic elastase. SPD, spermidine. OTUs, operational taxonomic units.

Given the dysfunction of SPD metabolism in the gut microbiota of the PPE group, we explored the therapeutic effect of exogenous SPD on AAA. Vehicle or SPD was administered via drinking water as detailed in Figure 3A. The AAA diameters in both two groups were comparable after PPE infusion but increased more slowly in the PPE-SPD group compared to those in the PPE group (Figure 3B,C and Supplemental Figure S2A,B). In addition, SPD alleviated AAA progression as evidenced by attenuating elastin destruction and vascular smooth muscle cells depletion via histopathological examination (Supplemental Figure S2C), which was consistent with our previous study [15].

After 16S rDNA analysis, 910 OTUs co-occurred between the PPE and PPE-SPD groups and exhibited higher relative abundance in the PPE-SPD group, which suggested SPD rescued the reduced relative abundance in AAA (Figure 3D–F). In contrast, 789 OTUs co-occurred and exhibited comparable relative abundance between the sham and SPD groups (Supplemental Figure S3A,B). Further analysis was conducted to investigate the different compositions of gut microbiota at the family/genus taxonomic level (Supplemental Figure S2D,E and Supplemental Figure S3D).

Since the aortic diameter is significantly related to the progression and rupture of AAA, the maximal aortic diameter remains a mainstay for the assessment of AAA disease [21]. Therefore, linear regression analysis was used to explore the correlation between AAA diameters and the gut microbiome at the family/genus level (Figure 3G). Among these bacterial genera, AAA diameters were positively associated with Parabacteroides, and negatively with Paraprevotellaceae, Prevotella and RF32. The Pearson’s correlation coefficients are shown in Supplemental Table S2 at each taxonomic level.

### 3.4. SPD Remitted Gut Microbiota Dysbiosis in Experimental AAA

**Figure 4:**
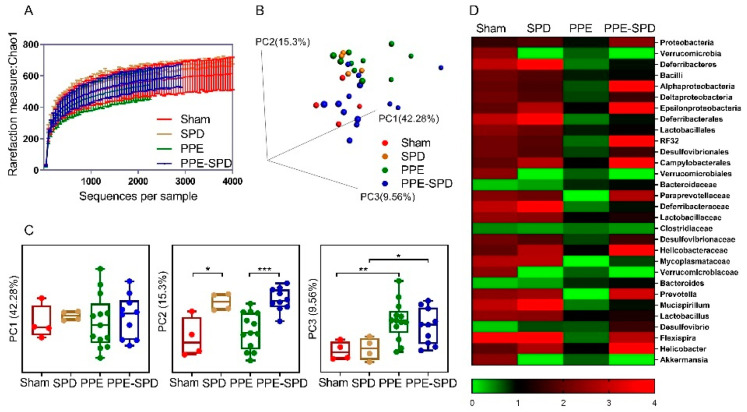
Gut microbiota diversity in four groups. (**A**) Alpha diversity analysis based on Chao 1 index of gut microbiota in sham group (n = 4), SPD group (n = 4), PPE group (n = 13) and PPE-SPD group (n = 10). (**B**,**C**) Principal component analysis (PCA) and ANOSIM analysis based on the Bray–Curtis distance in three groups. Significance was analyzed using one-way ANOVA and the Kruskal–Wallis test (* *p* < 0.05, ** *p* < 0.01, *** *p* < 0.001). In all box plots, boxes represent the interquartile ranges (IQRs) between the first and third quartiles, and the line inside the box represents the median. (**D**) Relative abundance of taxonomy in four groups. PPE, porcine pancreatic elastase. SPD, spermidine.

Next, we investigated the differences in gut microbiota modulation in four groups. The alpha diversity analysis based on the Chao 1 index revealed that gut microbiota diversity decreased after PPE infusion, and partially recovered by SPD administration, while no significant difference was observed between the sham and SPD groups (Figure 4A and Supplemental Figure S3C,D). In addition, a PCoA-3D analysis based on the Bray–Curtis distance was used to visualize the dissimilarity in the compositions of gut microbiota communities (Figure 4B). Although no difference was observed on PC1, it suggested that PPE-infusion and SPD exerted significant effects on community compositional structures on PC2 and PC3, respectively (Figure 4C). Furthermore, the relative abundance of taxonomy in four groups is shown in the heatmap (Figure 4D).

**Figure 5:**
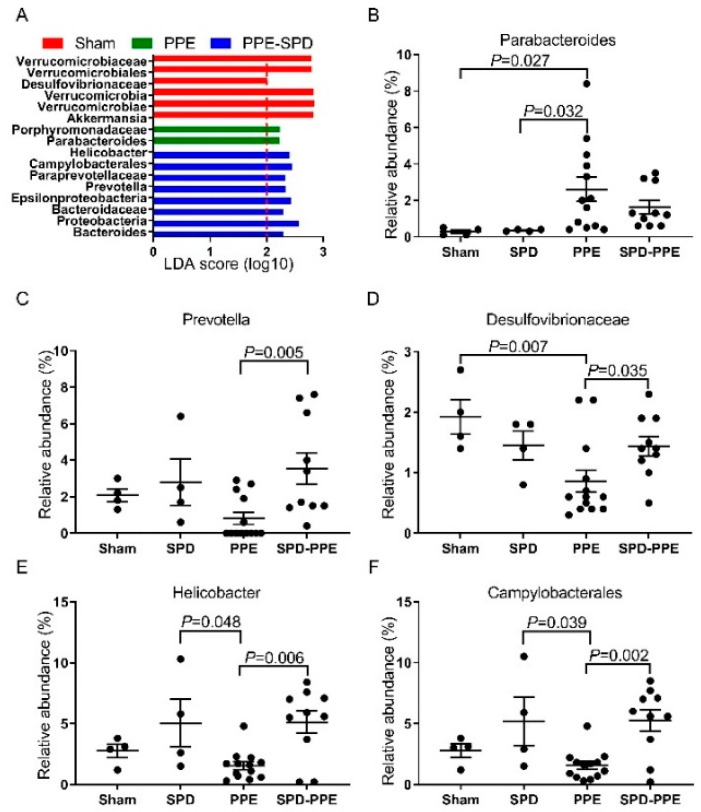
Abundant changes of gut flora in mice after PPE infusion and SPD treatment. (**A**) Histogram of the linear discriminant analysis (LDA) scores for significantly changed bacteria by LEfSe analysis in four groups. No bacteria with an LDA score ≥ 2 were identified in the SPD group. The related bacteria names of each column are listed on the left, and the score number is shown on the X-axis. (**B**–**F**) Relative abundances of differentially abundant taxons in each group. Data are presented as mean ± SEM and significance values were calculated using one-way ANOVA and Kruskal–Wallis test. PPE, porcine pancreatic elastase. SPD, spermidine.

Then, LEfSe analysis revealed the differences in gut microbiota among four groups and identified the differentially abundant taxons in each group (Figure 5A). As is shown in Figure 5B–F, the relative abundance of Paraprevotellaceae/Prevotella, Desulfovibrionaceae, Proteobacteria/Epsilonproteobacteria/Campylobacterales and Helicobacter decreased and Porphyromonadaceae/Parabacteroides increased after in PPE infusion, while SPD administration remitted these gut microbiota modulations. These results were consistent with the above data about the correlation between AAA diameters and the gut microbiome (Figure 3G, Table S2), which suggested that the therapeutic effect of SPD may be associated with remitted gut microbiota modulation in AAA.

### 3.5. SPD Restored KEGG Orthology and Metabolism of Gut Microbiome in Experimental AAA

**Figure 6:**
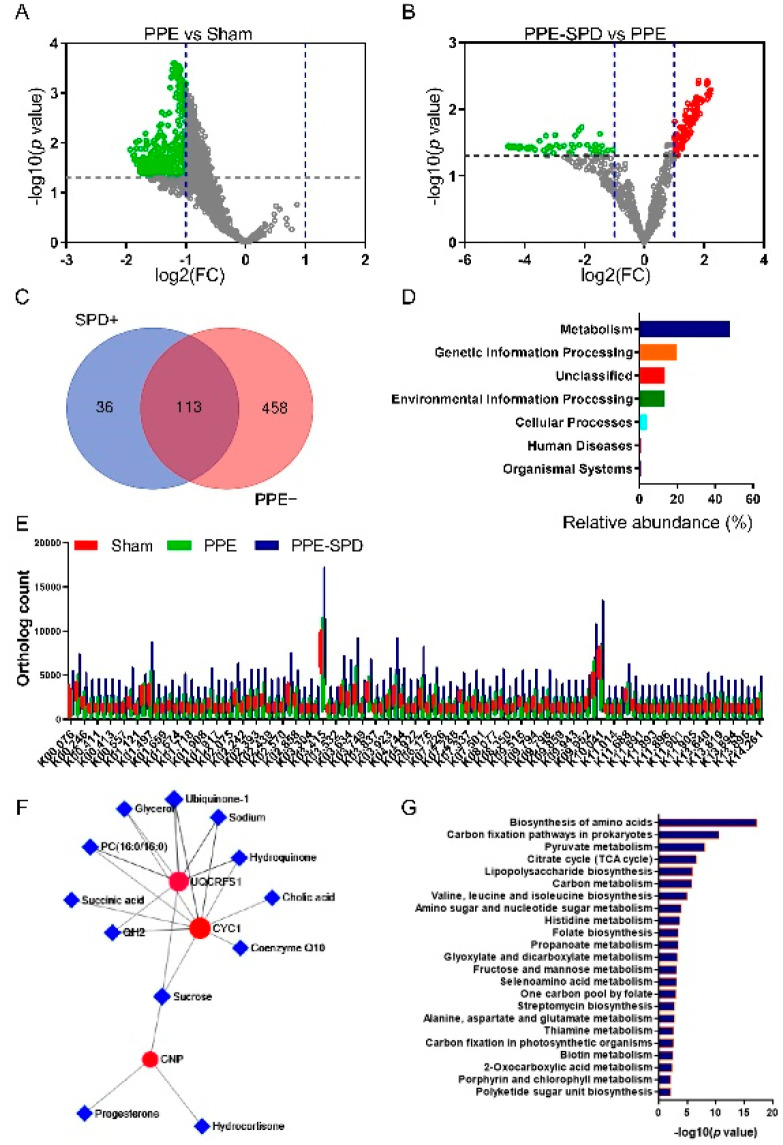
SPD restored KEGG Orthology and metabolism of gut microbiome in experimental AAA. (**A**) Volcano plot analyzing KOs in the PPE group compared with those in the sham group. (**B**) Volcano plot analyzing KOs in the PPE-SPD group compared with those in the PPE group. (**C**) Venn diagram of upregulated KOs in the PPE-SPD group and downregulated KOs in the PPE group. There were 113 KOs shared between the two groups. (**D**) Ortholog count of the 113 shared KOs. Data are presented as mean ± SEM. The name of indicated KOs and involved enzymic reactions are shown in supplemental Table S3. (**E**) Relative abundance of microbial KEGG modules. The related pathway annotations of KEGG pathway L1 are listed on the left. (**F**) KEGG network analysis and (**G**) enriched gene ontology terms of the significantly altered KOs. AAA, abdominal aortic aneurysm. KEGG, Kyoto Encyclopedia of Genes and Genomes. KOs, KEGG Orthologs. PPE, porcine pancreatic elastase. SPD, spermidine.

To gain further insights into functional changes in the gut microbiome in response to SPD treatment, we annotated genes to KEGG Orthology (KO). KO analysis demonstrated that 571 KOs were downregulated in the PPE groups compared to the sham group, while 149 KOs were upregulated in the PPE-SPD groups compared to the PPE group (Figure 6A,B). Of those, 113 KOs were recovered by SPD treatment in experimental AAA as shown in the Venn diagram (Figure 6C). The orthology counts of 113 KOs are listed in Figure 6E and Supplemental Table S3. The functional profile of the microbial community showed a significant difference in the predicted functions after SPD administration in AAA.

Next, GO analysis indicated that these 113 KOs were involved in 7 KEGG pathways at level 1, and the most upregulated pathway was that involved in metabolism (Figure 6D). The functional genes from the KEGG pathway sequence hierarchy level 1 (genes relevant to metabolism) were evaluated based on KEGG network analysis. As suggested in Figure 6F,G, the metabolic network analysis showed upregulation for UQCRFS1/CYC1 and CNP signal and enrichment for the biosynthesis of amino acids and carbon fixation pathways.

## 4. Discussion

By using 16S rDNA sequencings, we explored the alteration to gut microbiome in PPE-infused experimental AAA mice and the effect of SPD administration on the gut microbiome in the background of AAA disease. In the present study, AAA implicated a remarkable gut dysbiosis characterized by an altered gut microbial profile and metabolism. In addition, exogenous SPD remitted gut microbiota modulation and metabolism in AAA. Thus, these findings prompted us to determine whether gut microbiota dysbiosis contributes to AAA development and how SPD protects gut microbiota function in AAA.

Mounting preclinical and clinical research has highlighted the critical role of the gut microbiome and intestinal barrier function in cardiovascular and metabolic disease, which has led to the concept of the “leaky gut” [22,23]. Intestinal permeability, regulated by gut microbiota modifications, is a feature of intestinal barrier function and exerts significant effects on health and disease [24]. Most evidence confirms that viable organisms colonize in aortic aneurysms which may be attributed to the oral cavity and gastrointestinal tract [3,7,25,26]. Although the role of the gut microbiome in modulating intestinal permeability is not completely understood, credible evidence has reported that increased intestinal permeability promotes vasculitis and AAA in Kawasaki disease mouse models [27]. Moreover, our previous study also reported that oral administration of Akkermansia muciniphila could restore diversity to the gut microbiota, regulate the functional pathways related to Lactobacillus and l-rhamnose degradation/synthesis and inhibit CaCl2-induced experimental AAA formation [28]. Therefore, the direct roles of gut microbiome that participate in AAA expansion are predicted.

Our results suggested that gut microbiome diversity decreased in AAA characterized by lower alpha and beta diversity. Of these, Bacteroides, within the Bacteroidaceae family, were most obviously elevated in this study. Bacteroides, anaerobic Gram-negative bacteria, generally colonize the human and animal gastrointestinal tract. The Bacteroides species can function as an integral partner in the human metabolic system and, yet, can be the cause of a variety of diseases such as chronic infectious disorders and inflammatory diseases [29]. It has also been demonstrated that Bacteroides are associated with susceptibility to autoimmune diseases and atherosclerosis [30,31]. Interestingly, a bacterial community of Bacteroides species was over-represented in intracranial aneurysm individuals and mice, which indicates its potential effect on the progression of unruptured intracranial aneurysms [32]. Moreover, a recent case report describes the direct invasion and colonization of Bacteroides dorei in human AAA tissues providing a warning regarding the dysbiosis of Bacteroides in AAA disease [33]. In addition, decreased Oscillospira and Ruminococcus were observed in experimental AAA. Oscillospira and Ruminococcus are bacterial genera in the class Clostridia which are involved in the formation of common core bacteria in the western adult population. Oscillospira is previously reported to be essential for balancing gut microbes to protect against inflammatory bowel disease and cardiovascular disease [34,35]. Our results demonstrated a negative correlation between Oscillospira and AAA which suggested the potential mechanisms of probiotic involvement in AAA. Conversely, Ruminococcus species were connected to cardiovascular phenotypes in obese individuals and positively associated with angiotensin II-induced aortic aneurysms [36,37]. This may be explained by the different pathology among the two experimental AAA models considering dysregulated lipid metabolism in angiotensin II-induced aneurysm.

We also observed functional changes in the gut microbiome in AAA characterized by decreased biosynthesis and transport of polyamine. Reduced ortholog counts of SpeA/E4.1.1.19 and speE, encoding arginine decarboxylase and SPD synthase, respectively, were observed in experimental AAA, which suggested polyamine metabolism dysbiosis in the gut microbiota might be involved in AAA [38]. Polyamine, especially SPD, was reported to correlate with reduced cardiovascular and cancer-related mortality [13]. SPD presents in the gastrointestinal tract with different origins: de novo biosynthesis, diet and luminal bacteria [39]. Although previous studies have demonstrated the protective role of SPD in cardiovascular disease including AAA, the relationship between SPD intake and the effect on gut microbiota remains unclear [13,15].

Our results suggested SPD suppressed experimental AAA in association with remitting the dysbiosis of gut microbiota modulation and metabolism. Porphyromonadaceae/Parabacteroides were identified to be upregulated in AAA and positively associated with AAA diameters, while Paraprevotellaceae/Prevotella showed a reversed trend. SPD administration partially normalized the dysbiosis of these gut microbiotas. The genus Parabacteroides is a group of Gram-negative anaerobic bacteria that commonly colonize the gastrointestinal tract of numerous species, which have ambivalent effects on human health and disease. Despite the lack of direct evidence between Parabacteroides and AAA disease, an increased abundance of Parabacteroides was observed in cardiovascular diseases such as atherosclerosis and hypertension [30,40,41]. Parabacteroides were also significantly over-represented in patients with unruptured intracranial aneurysms, which indicates its potential effect on AAA [32]. Prevotella, at the family of Paraprevotellaceae, is abundant in the digestive tract of people with a fiber-rich or Mediterranean diet [42]. Prevotella genus are considered commensal microbes due to their extensive abundance and low pathogenicity in the healthy human body [43]. Previous evidence revealed beneficial effects of some Prevotella strains improving CVD risk factor profile and autoimmune diseases [43,44,45]. This research indicated that the protective effect of SPD on AAA might be associated with remission of Prevotella dysbiosis. In addition, Desulfovibrionaceae was also found to be significantly decreased in AAA and remitted by SPD treatment in the present study. Family Desulfovibrionaceae was first proposed by Kuever et al. (2005) and showed a polyamine pattern with the predominant SPD. Recent studies reported that IgA-bound bacteria and Ranitidine could mitigate high-fat-diet-induced intestinal barrier damage and cardiovascular damage, mainly by stimulating an increase in Desulfovibrionaceae [46]. This showed that Desulfovibrionaceae might play a role in maintaining intestinal barrier function and exhibiting potential positive effects in this study. The functional profile of microbial community showed the effect of SPD was mainly related to metabolism, including amino acids, carbon fixation, pyruvate and citrate cycle. It supports the notion that SPD exerted an influence on gut microbiota metabolism and promoted intestinal epithelial repair and barrier function in diet-induced obesity [14,47]. Together, these results revealed the potential effect of SPD on gut microbiota modulation and intestinal barrier function in the progression of AAA.

Dietary SPD uptake exerts antiaging, neurologic and cardiovascular benefits with no apparent adverse reactions in long-term use in mice and humans [13,18,48,49]. Upregulation of SPD by dietary and gut microbiota can help compensate for the aging-associated declines in SPD through intestinal lumen, thereby extending the healthy life expectancy. Although an in vivo mechanism has not been fully elucidated, these recent studies suggest that gut microbiota might play a role in the therapeutic effect of SPD. It has been reported that the interaction of spermidine and gut microbiota contributes to fine-tuning the intestinal mucosal epithelium and immunity, which ultimately maintains mucosal homeostasis and delays senescence in mice [50,51]. Furthermore, a recent study reported mice with colitis exhibited dysbiosis with a reduced abundance of Prevotella and increased Proteobacteria and Deferribacteres, which was reversed by oral SPD [52]. Similar effects of SPD can also be seen in heart failure [53] and nonalcoholic steatohepatitis [54]. Together, our current findings and previous studies demonstrate the important role of SPD in influencing microbiota composition and resilience, which exert therapeutic effects on various diseases. SPD is safe and well tolerated in older adults and mice [48], so it is being applied for multiple clinical trials and might be very promising in clinical application.

Some limitations of our study need to be acknowledged. First, the number of mice included in the sham group is relatively small, so the statistical power of the study is limited. Due to the difficulty of PPE-induced AAA model surgery [55], it limits the number of mice in each group. Our results demonstrated the heterogeneity of the sham group seemed to be lower compared to other groups (see PCoA analysis in Figure 4B,C). Therefore, we preferred to increase the number of mice in the PPE group and the PPE-SPD group, which may contribute to demonstrating gut microbiome modulation in the progression of AAA disease. Interestingly, our results demonstrated that these bacterial genera associated with AAA diameters, such as Parabacteroides and Prevotella, were identified as the differentially abundant taxons after PPE infusion. It suggested the number of mice in the sham group might not affect the main findings of the study. Second, we selected the cecal contents instead of stools sample as the study object to evaluate gut microbiota modulation, which eliminated the opportunity of having a baseline microbiome profile before the experimental interventions. This is because polyamine is mainly absorbed in the small intestine and formed by gut microbiota primarily in the large intestine [10]. The intestinal contents might be more helpful to elucidate the relationship between polyamine and gut microbiome modulation.

## 5. Conclusions

Our results uncovered altered gut microbiota profiles and function in AAA, with increased Bacteroides and Parabacteroides and reduced Prevotella, Desulfovibrionaceae, as well as dysregulating the biosynthesis and transportation of SPD. Exogenous SPD remitted the gut microbiota dysbiosis and attenuated the progression of AAA, which highlights the potential therapeutic use of SPD in the treatment of gut microbiota dysbiosis and AAA. SPD supplementation via an SPD-enriched diet is safe and well-tolerated in humans, making it possible to offer a cost-effective and reasonable alternative therapy for AAA and other diseases.

## Supplementary Materials

The following supporting information can be downloaded at: https://www.mdpi.com/article/10.3390/nu14163349/s1, Figure S1: The histopathology and gut microbiota modulation in PPE-induced AAA disease; Figure S2: Effect of SPD on AAA progression and gut microbiota modulation; Figure S3: Alterations in gut microbiota between the sham group and the SPD group; Table S1: Enzyme annotation in polyamine metabolism using the KEGG Orthology; Table S2: The Pearson’s correlation coefficients at each taxonomic level between AAA diameters and the gut microbiome; Table S3: KEGG Orthology-based annotation of the significantly altered KOs.
