# Supplementary material for: Effects of Spermidine on Gut Microbiota Modulation in Experimental Abdominal Aortic Aneurysm Mice
Source: Nutrients. 2022 Aug 16;14(16):3349. doi: 10.3390/nu14163349 (PMC9415871; doi:10.3390/nu14163349)
Supplement: Supplementary file 1 [file nutrients-14-03349-s001.zip › Supplementary Table S1.pdf]

Table S1 Enzyme annotation in polyamine metabolism using the KEGG Orthology

KEGG_Entry	Entry_Desc
K00,797	speE;spermidine synthase [EC:2.5.1.16]
K01,480	speB;agmatinase [EC:3.5.3.11]
K01,583	E4.1.1.19;arginine decarboxylase [EC:4.1.1.19]
K01,585	speA;arginine decarboxylase [EC:4.1.1.19]
K01,611	speD;S-adenosylmethionine decarboxylase [EC:4.1.1.50]
K02,052	ABC.SP.A;putative spermidine/putrescine transport system ATP-binding protein
K02,053	ABC.SP.P;putative spermidine/putrescine transport system permease protein
K02,054	ABC.SP.P1;putative spermidine/putrescine transport system permease protein
K11,069	potD;spermidine/putrescine transport system substrate-binding protein
K11,070	potC;spermidine/putrescine transport system permease protein
K11,071	potB;spermidine/putrescine transport system permease protein
K11,072	potA;spermidine/putrescine transport system ATP-binding protein [EC:7.6.2.11]
K11,742	mdtI;spermidine export protein MdtI
K11,743	mdtJ;spermidine export protein MdtJ
K13,747	nspC;carboxynorspermidine decarboxylase [EC:4.1.1.96]
