# Supplementary material for: Effects of Spermidine on Gut Microbiota Modulation in Experimental Abdominal Aortic Aneurysm Mice
Source: Nutrients. 2022 Aug 16;14(16):3349. doi: 10.3390/nu14163349 (PMC9415871; doi:10.3390/nu14163349)
Supplement: Supplementary file 1 [file nutrients-14-03349-s001.zip › Supplementary Table S2.pdf]

Table S2. The Pearson's correlation coefficients at each taxonomic level between AAA diameters and the gut microbiome

	#OTU ID	Pearson *r*	*P* value
Order	RF32	−0.4147	0.0491
Family	Paraprevotellaceae	−0.4875	0.0183
	Porphyromonadaceae	0.4583	0.0279
Genus	Prevotella	−0.4558	0.0288
	Parabacteroides	0.4583	0.0279
