# Supplementary material for: Effects of Spermidine on Gut Microbiota Modulation in Experimental Abdominal Aortic Aneurysm Mice
Source: Nutrients. 2022 Aug 16;14(16):3349. doi: 10.3390/nu14163349 (PMC9415871; doi:10.3390/nu14163349)
Supplement: Supplementary file 1 [file nutrients-14-03349-s001.zip › Supplementary Table S3.pdf]

Table S3 KEGG Orthology-Based Annotation of the significantly altered KOs

KEGG_Entry	Entry_Desc
K00,076	7-alpha-hydroxysteroid dehydrogenase [EC:1.1.1.159]
K00,124	formate dehydrogenase, beta subunit [EC:1.2.1.2]
K00,246	fumarate reductase subunit C; fumarate reductase subunit C [EC:1.3.99.1]
K00,407	cytochrome c oxidase cb-type subunit IV; cb-type cytochrome c oxidase subunit IV [EC:1.9.3.1]
K00,411	ubiquinol-cytochrome c reductase iron-sulfur subunit [EC:1.10.2.2]
K00,412	ubiquinol-cytochrome c reductase cytochrome b subunit; ubiquinol-cytochrome c reductase cytochrome b subunit [EC:1.10.2.2]
K00,413	ubiquinol-cytochrome c reductase cytochrome c1 subunit [EC:1.10.2.2]; ubiquinol-cytochrome c reductase cytochrome c1 subunit
K00,429	catalase [EC:1.11.1.6]
K00,557	tRNA (uracil-5-)-methyltransferase [EC:2.1.1.35]
K00,619	amino-acid N-acetyltransferase [EC:2.3.1.1]
K01,121	2',3'-cyclic-nucleotide 3'-phosphodiesterase [EC:3.1.4.37]
K01,139	guanosine-3',5'-bis(diphosphate) 3'-pyrophosphohydrolase [EC:3.1.7.2]
K01,497	GTP cyclohydrolase II [EC:3.5.4.25]
K01,514	exopolyphosphatase [EC:3.6.1.11]
K01,659	2-methylcitrate synthase [EC:2.3.3.5]
K01,664	para-aminobenzoate synthetase component II [EC:2.6.1.85]
K01,674	carbonic anhydrase [EC:4.2.1.1]
K01,682	aconitate hydratase 2 [EC:4.2.1.3]
K01,718	pseudouridylate synthase [EC:4.2.1.70]
K01,720	2-methylcitrate dehydratase [EC:4.2.1.79]
K01,908	propionyl-CoA synthetase [EC:6.2.1.17]
K01,916	NAD+ synthase [EC:6.3.1.5]
K01,917	glutathionylspermidine synthase [EC:6.3.1.8]

K01,959	pyruvate carboxylase subunit A [EC:6.4.1.1]
K02,075	zinc/manganese transport system permease protein
K02,195	heme exporter protein C
K02,242	competence protein ComFC
K02,386	flagella basal body P-ring formation protein FlgA
K02,393	flagellar L-ring protein precursor FlgH
K02,404	flagellar biosynthesis protein FlhF
K02,439	thiosulfate sulfurtransferase [EC:2.8.1.1]
K02,568	cytochrome c-type protein NapB
K02,570	periplasmic nitrate reductase NapD
K02,574	ferredoxin-type protein NapH
K02,858	3,4-dihydroxy 2-butanone 4-phosphate synthase [EC:4.1.99.12]
K03,191	acid-activated urea channel
K03,304	tellurite resistance/dicarboxylate transporter, TDT family
K03,342	para-aminobenzoate synthetase / 4-amino-4-deoxychorismate lyase [EC:2.6.1.85 4.1.3.38]
K03,415	two-component system, chemotaxis family, response regulator CheV
K03,417	methylisocitrate lyase [EC:4.1.3.30]
K03,532	trimethylamine-N-oxide reductase(cytochrome c) 1,cytochrome c-type subunit TorC
K03,605	hydrogenase 1 maturation protease [EC:3.4.24.-]
K03,634	outer membrane lipoprotein carrier protein
K03,694	ATP-dependent Clp protease ATP-binding subunit ClpA
K03,749	DedD protein
K03,833	selenocysteine-specific elongation factor
K03,837	serine transporter
K03,893	arsenical pump membrane protein
K03,923	modulator of drug activity B
K03,981	thiol:disulfide interchange protein DsbC [EC:5.3.4.1]

K04,744	LPS-assembly protein
K05,835	threonine efflux protein
K05,922	quinone-reactive Ni/Fe-hydrogenase large subunit [EC:1.12.5.1]
K05,927	quinone-reactive Ni/Fe-hydrogenase small subunit [EC:1.12.5.1]
K06,176	tRNA pseudouridine13 synthase [EC:5.4.99.27];<br>tRNA pseudouridine synthase D [EC:5.4.99.12]
K06,603	flagellar protein FlaG
K07,226	hypothetical protein
K07,270	glycosyl transferase, family 25
K07,288	uncharacterized membrane protein
K07,318	adenine-specific DNA-methyltransferase [EC:2.1.1.72]
K07,337	hypothetical protein
K07,457	endonuclease III related protein
K07,501	hypothetical protein
K07,710	two-component system, NtrC family, sensor histidine kinase AtoS [EC:2.7.13.3]
K08,077	UDP-sugar diphosphatase [EC:3.6.1.45]
K08,306	membrane-bound lytic murein transglycosylase C [EC:3.2.1.-]
K08,350	formate dehydrogenase-N, gamma subunit [EC:1.2.1.2]
K08,973	putative membrane protein
K09,516	all-trans-retinol 13,14-reductase [EC:1.3.99.23]
K09,726	hypothetical protein
K09,794	hypothetical protein
K09,795	hypothetical protein
K09,798	hypothetical protein
K09,804	hypothetical protein
K09,859	hypothetical protein
K09,860	hypothetical protein
K09,943	hypothetical protein

K09,944	hypothetical protein
K09,952	hypothetical protein
K10,040	putative glutamine transport system permease protein
K10,041	putative glutamine transport system ATP-binding protein [EC:3.6.3.-]
K11,013	cytolethal distending toxin subunit A
K11,014	cytolethal distending toxin subunit B
K11,015	cytolethal distending toxin subunit C
K11,688	C4-dicarboxylate-binding protein DctP
K11,742	spermidine export protein MdtI
K11,891	type VI secretion system protein ImpL
K11,892	type VI secretion system protein ImpK
K11,893	type VI secretion system protein ImpJ
K11,895	type VI secretion system protein ImpH
K11,896	type VI secretion system protein ImpG
K11,900	type VI secretion system protein ImpC
K11,901	type VI secretion system protein ImpB
K11,903	type VI secretion system secreted protein Hcp
K11,905	type VI secretion system protein
K11,906	type VI secretion system protein VasD
K13,640	MerR family transcriptional regulator, heat shock protein HspR
K13,797	DNA-directed RNA polymerase subunit beta-beta' [EC:2.7.7.6]
K13,819	NifU-like protein
K13,893	microcin C transport system substrate-binding protein
K13,894	microcin C transport system permease protein
K13,895	microcin C transport system permease protein
K13,896	microcin C transport system ATP-binding protein
K14,048	urease subunit gamma/beta [EC:3.5.1.5]
K14,261	alanine-synthesizing transaminase [EC:2.6.1.-]
