# Supplementary material for: Effects of Spermidine on Gut Microbiota Modulation in Experimental Abdominal Aortic Aneurysm Mice
Source: Nutrients. 2022 Aug 16;14(16):3349. doi: 10.3390/nu14163349 (PMC9415871; doi:10.3390/nu14163349)
Supplement: Supplementary file 1 [file nutrients-14-03349-s001.zip › Supplymental Figures S1-S3pdf.pdf]

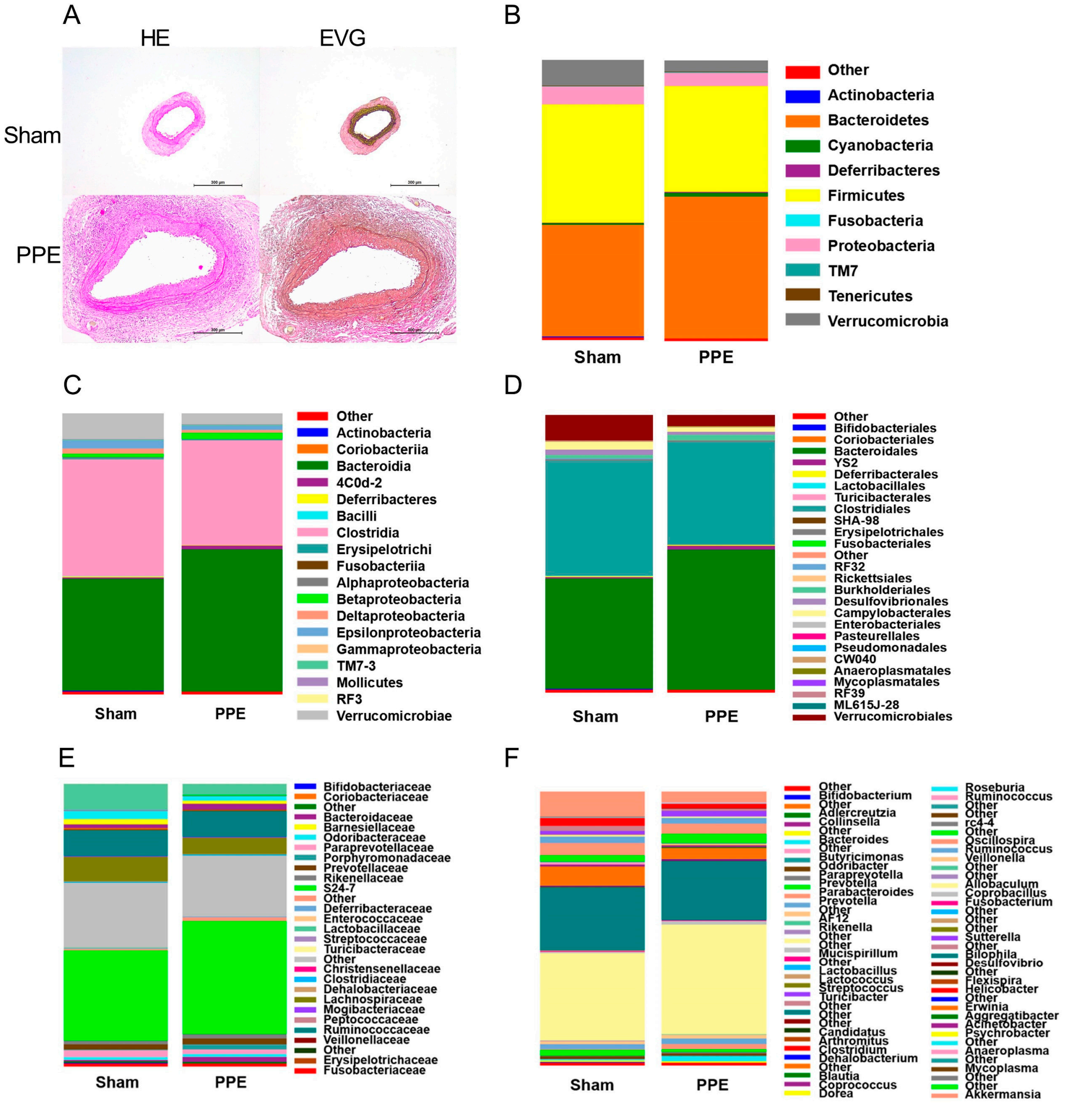

Figure S1. The histopathology and gut microbiota modulation in PPE-induced AAA disease (A) Representative aortic histology images of H&E and EVG staining in Sham and PPE group. (B) OTUs of gut microbiota at the phylum/order/class/family/genus taxonomic level in Sham group and PPE group.

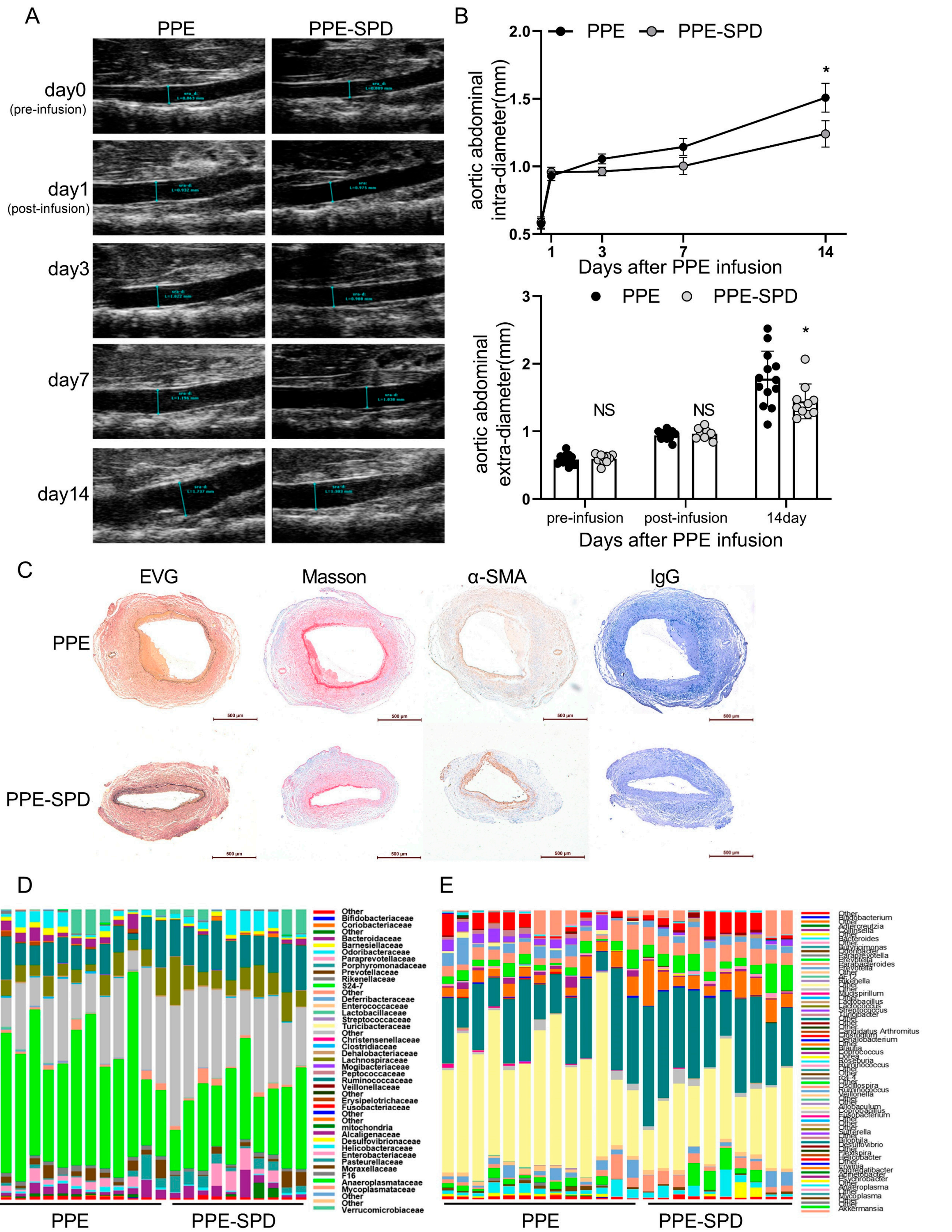

Figure S2. Effect of SPD on AAA progression and gut microbiota modulation

(A) Ultrasound images of abdominal aorta of PPE and PPE-SPD group. (B) intra-diameter and extra-diameter for the experimental AAA models of PPE group (n=13) and PPE-SPD group (n=10). Data were presented as mean ± SEM. Significance was analyzed using two-way ANOVA with the Bonferroni correction and Unpaired *t* test respectively. $^*P < 0.05$, vs. PPE group. (C) Representative aortic histology images of EVG, Masson's, and SMC α actin staining in PPE and PPE-SPD group. IgG staining serves as the negative control. (D) OTUs of gut microbiota at the family/genus taxonomic level in PPE group and PPE-SPD group.

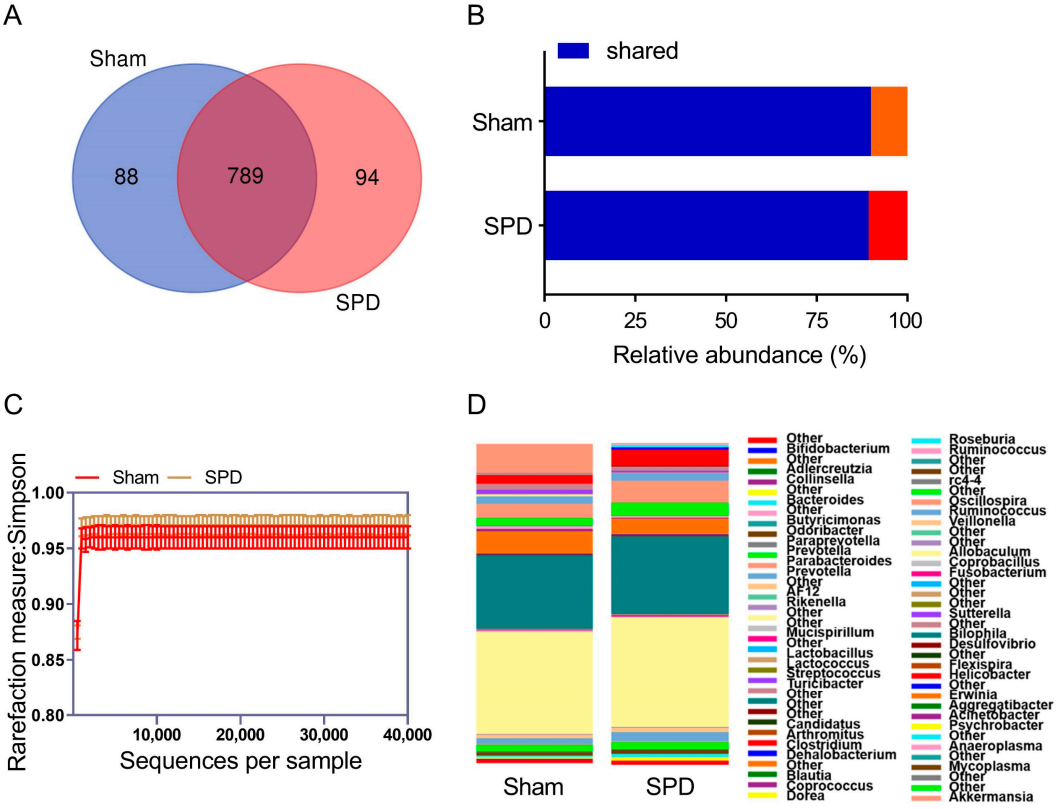

Figure S3. Alterations in gut microbiota between Sham group and SPD group

(A) The number of OTUs in Sham group, SPD group and shared OTUs between two groups in a scaled Venn diagram. (B) Total abundance of OTUs in two groups. (C) Alpha diversity measured by Simpson rarefaction curve. (D)OTUs of gut microbiota at the genus taxonomic level in Sham group and SPD group.
